# Hypertension Control Cascade and Regional Performance in India: A Repeated Cross-Sectional Analysis (2015-2021)

**DOI:** 10.7759/cureus.35449

**Published:** 2023-02-25

**Authors:** Saurav Basu, Mansi Malik, Tanu Anand, Angad Singh

**Affiliations:** 1 Medicine, Indian Institute of Public Health - Delhi, Public Health Foundation of India, New Delhi, IND; 2 Clinical Research, Indian Institute of Public Health-Delhi, Public Health Foundation of India, New Delhi, New Delhi, IND; 3 Medicine, Indian Council of Medical Research, Delhi, IND; 4 Biostatistics, International Institute for Population Sciences, Mumbai, IND

**Keywords:** nfhs, survey research, drug access, medication adherence, blood pressure control, hypertension

## Abstract

Background

The weak control cascade of hypertension from the time of screening till the attainment of optimal blood pressure (BP) control is a public health challenge, particularly in resource-limited settings. The study objectives were to (1) estimate the change in the rate of prevalence of hypertension, the yield of newly diagnosed cases, initiation of treatment, and attainment of BP control in the age group 15 to 49 years; (2) ascertain the magnitude and predictors of undiagnosed hypertension, lack of initiation of treatment, and poor control of those on antihypertensive therapy; and (3) estimate the regional variation and state-level performance of the hypertension control cascade in India.

Methodology

We analyzed demographic and health surveillance (DHS) data from India’s National Family Health Survey Fifth Series (NFHS-5), 2019-2021, and NFHS-4 (2015-2016). The NFHS-5 sample comprised 695,707 women and 93,267 men in the age group of 15 to 49 years. Multiple logistic regressions were performed to find the associated predictors, and respective adjusted odds ratios (aORs) were reported.

Results

The prevalence of hypertension (cumulative previously diagnosed and new cases) among individuals aged 15 to 49 years was 22.8% (22.6%, 23.1%; *n* = 172,532), out of which 52.06% were newly diagnosed cases. In contrast, in NFHS-4, the prevalence of hypertension among individuals aged 15 to 49 years was 20.4% (20.2%, 20.6%; *n* = 153,384), of which 41.65% were newly diagnosed cases.

In NFHS-5, 40.7% (39.8% and 41.6%) of the previously diagnosed cases were on BP-lowering medications compared to 32.6% (31.8%, 33.6%) in NFHS-4. Furthermore, in NFHS-5, controlled BP was observed in 73.7% (72.7% and 74.7%) of the patients on BP-lowering medication compared to 80.8% (80.0%, 81.6%) in NFHS-4. Females compared to males (aOR = 0·72 and 0·007), residents of rural areas (aOR = 0·82 and 0·004), and those belonging to the socially disadvantaged groups were not initiated on treatment despite awareness of their hypertension status indicative of poor treatment-seeking behavior. Furthermore, increasing age (aOR = 0·49, *P *< 0·001), higher body mass index (aOR = 0·51, *P *< 0·001), and greater waist-to-hip ratio (aOR = 0·78, *P *= 0·047) were associated with uncontrolled hypertension in patients on antihypertensive drug therapy.

Conclusions

Hypertension control cascade in India is largely ineffectual although screening yield and initiation of antihypertensive treatment have improved in NFHS-5 compared to NFHS-4. Identification of high-risk groups for opportunistic screening, implementing community-based screening, strengthening primary care, and sensitizing associated practitioners are urgently warranted.

## Introduction

Hypertension is a major cause of cardiovascular disease and deaths worldwide, especially in low- and middle-income countries (LMICs). In 2019, around 1.28 billion adults aged between 30 and 79 years are estimated to be affected by hypertension worldwide, with the prevalence of hypertension being 32% among women and 34% among men [1,2]. As per the global burden of diseases 2019 estimates, hypertensive heart disease considering all ages and sexes accounts for 0.85% of total Disability Adjusted Life Years (DALYs) globally and has shown an upward trend [3]. The aging of the population and increased exposure to lifestyle risk factors, such as unhealthy diets (high salt and low potassium intake) and lack of physical activity, contribute to the increase [4]. In India, too, the estimated number of DALYs associated with hypertension has increased from 21 million in 1990 to 39 million in 2016 (+89%) [5]. The prevalence of hypertension as per the National Family Health Survey Series Four (NFHS-4) was found to be 18.1% in 2015-2016 [6], while 21% of females aged over 15 years had hypertension compared to 24% of males of the same age range, as estimated in NFHS-5 (2019-2021) [7].

According to the *rule of halves* for hypertension, *half the people with high blood pressure (BP) are not known* (rule 1), *half of those who are known are not treated* (rule 2), and *half of those who are treated are not controlled* (rule 3) [8]. The weak care cascade of hypertension from the time of screening, diagnosis, treatment initiation, and the attainment of optimal BP control is thereby a public health challenge, particularly in resource-limited settings. Consequently, despite the availability of safe, well-tolerated, and cost-effective BP (BP)-lowering therapies, <14% of adults with hypertension have BP controlled to a systolic BP (SBP)/diastolic BP (DBP) <140/90 mmHg [1].

The secondary data analysis of NFHS-4 revealed that among patients with hypertension in India, only 63.2% had their BP measured earlier, while only 21.5% were aware of their diagnosis [6]. In another repeated cross-sectional survey in the National Capital Region of India, among 3,048 individuals, the prevalence of hypertension was reported to have increased over 20 years with no improvement in its management [9]. Untreated hypertension or resistant hypertension can substantially increase the chances of heart attack, stroke, and kidney failure [10]. Also, long-standing and uncontrolled hypertension is a strong risk factor for microvascular and macrovascular complications such as ischemic heart disease, stroke, chronic kidney disease, retinopathy, etc. [11].

Only a few studies have explored the care cascades and treatment-seeking behavior of patients with hypertension in India [9]. Furthermore, a comparison of state health performance with treatment-seeking and hypertension control has not been assessed previously in Indian health settings. These data are pertinent to inform policy and programs for hypertension control in India. Consequently, analysis of nationally representative empirical data for understanding the existing barriers and challenges in the hypertension control cascade in India and ways of strengthening the same through a focus on effective public health interventions is urgently warranted. The study objectives were to estimate in the age group of 15-49 years in India (1) the change in the rate of prevalence of hypertension, the yield of newly diagnosed cases, initiation of treatment, and attainment of BP control; (2) ascertain the magnitude and predictors of undiagnosed hypertension, lack of initiation of treatment, and poor control of those on antihypertensive therapy. Additionally, we evaluate regional estimates of the hypertension control cascade and compared them state-wise after stratifying them with a comprehensive health system performance index.

## Materials and methods

Data source and study population

The study was carried out on demographic and health surveillance (DHS) data from India’s NFHS-5 (2019-2021) and NFHS-4 (2015-2016) for comparative analysis. NFHS surveys provide data on India’s population and health for 707 districts, 28 states, and eight union territories. NFHS-5 is a two-stage stratified sample. Primary sampling units (PSUs) were villages in rural areas, and Census Enumeration Blocks (CEBs) in urban areas, and these PSUs were selected based on the probability proportional to size (PPS) sampling method. NFHS-5 included a sample of 788,974 participants, while NFHS-4 consisted of a sample of 770,783 participants. Men and women questionnaires collected information from candidates aged 15-54 and 15-49 years, respectively. Two sets of questionnaires (district and state module) were used for women while men had just one questionnaire (state module only) [12]. In this analysis, information was collected from a sample of men and women aged 15-49 years whose BP information was available in the biomarker dataset. We excluded men aged >49 years and pregnant women in this analysis.

Measurement of BP

All participants aged 15 years or more had their BP measured three times, with a five-minute gap between readings, using an Omron BP monitor (OMRON, Kyoto, Japan) [12].

Outcome variables and operational definitions

Hypertensive

We excluded the initial BP reading and calculated the average of the last two BP readings in the dataset. Individuals detected with average SBP >= 140 mmHg or DBP >= 90 mmHg on screening, or were previously told they had hypertension on two or more occasions (by a healthcare professional), or were taking antihypertensive medication were classified as hypertensive.

New Cases

New cases of hypertension were defined as individuals detected with hypertension on screening and responded *no* to the following two statements: (1) told they had high BP on two or more occasions by the doctor or other health professionals, and (2) currently taking prescribed medicine to lower BP. 

Awareness of Hypertension

Individuals who responded *yes* to the following statement were considered as being aware of their hypertensive status: *Told had high BP on two or more occasions by the doctor or other health professionals*.

On Hypertension Treatment

Individuals who responded *yes* to the following statement were considered to be on hypertension treatment: *Currently taking a prescribed medicine to lower BP*.

Controlled Hypertension

Individuals who were currently taking antihypertensive medication and were detected with SBP < 140 mmHg and DBP < 90 mmHG on screening were classified as having controlled hypertension.

Uncontrolled Hypertension

Individuals who were currently taking antihypertensive medication and were detected with SBP >= 140 mmHg or DBP >= 90 mmHg on screening were classified as having uncontrolled hypertension.

Independent variables

The predictor variables were selected based on literature reviews such as age, gender, education level (no education, primary, and secondary or higher education), place of residence (urban or rural), religion (Hindu, Muslim, Christian, or Others), lifestyle factors (smoking and alcohol), and marital status. The frequency of having fried food and aerated drinks was categorized as less frequent (never or occasionally) and more frequent (weekly or daily), presence of comorbidities such as diabetes and heart disease, wealth index, etc., were also considered. The healthcare facility was categorized into three groups: all public facilities as *Public*, all private facilities as *Private*, and nongovernmental organization (NGO) along with other facilities as *Other*.

Subgroup analysis

The regional variation in the prevalence and control of hypertension in India was estimated in all the states and union territories of India, wherein the outcomes were stratified as those individuals who were aware of their hypertensive condition and were on antihypertensive medication. Individuals were considered as having good control of their BP if SBP < 140 mmHg and DBP < 90 mmHg. The states of India were categorized as per their National Health Index score for the year 2019-2020, which classified them as High (HI score > 55), medium (HI score 45 to 55), and low (HI score < 45) [13]. Since 2017, the National Institution for Transforming India (NITI) Aayog, the apex public policy thinktank of the government of India has been leading the health index program to assess the annual performance of states and union territories on several metrics such as governance, procedures, and health results, although it includes predominant maternal and child health-related indicators as a proxy for the overall health status [14].

Data and statistical analysis

All the values of the variables were checked for their plausibility. Individual Men (IAMR7AFL) and Individual Women (IAIR7ADT) files were used for this analysis because the wide range of predictors incorporated in the study was not available in household files (e.g., type of healthcare facility utilized). Hence, the total sample size was 770,783 (women and men) in NFHS-4, while 788,974 (695,707 women and 93,267 men) in NFHS-5. There were several improbable values in the body mass index (BMI) of individuals, for which we replaced those with missing values, i.e., BMI values >80 and <7 were set as *missing values*. Appropriate weights were applied throughout the analysis for calculating the adjusted proportions and their 95% confidence interval (CI). Variables with a statistically significant association in bivariate analysis were included in the regression model. Multiple logistic regression was performed to find the predictors for hypertension awareness, treatment seeking, and its control. All the assumptions and prerequisites were checked for the logistic regression analysis. Predictor variables were assessed for multicollinearity. Toward the end, the model was assessed for its fitness. The analysis was performed in STATA version 15.1 (Stata Corp., College Station, TX, USA).

Ethical considerations

This study is a secondary data analysis of publicly available NFHS-5 data. All the respondents who took the survey provided their voluntary written and informed consent. NFHS-5 received ethical clearance from the ethical review board of the International Institute of Population Sciences (IIPS), Mumbai, India. Permission was also obtained from IIPS to conduct this analysis.

## Results

We analyzed a sample of 695,707 women and 93,267 men from the NFHS-5 data set to evaluate the prevalence of hypertensive individuals, awareness of their hypertensive status, and use of medication among aware individuals. Furthermore, we estimated the prevalence of uncontrolled hypertension among those taking the medication and the determinants of uncontrolled hypertension.

The baseline characteristics of the study population segregated by previously diagnosed and newly diagnosed cases are reported in Table 1. In NFHS-5, a total of 82,718 previously diagnosed hypertension cases and 89,814 new hypertension cases were detected on screening. The prevalence of hypertension (cumulative previously diagnosed and new cases) among individuals aged 15 to 49 years was 22.8% (22.6%, 23.1%; *n* = 172,532), out of which 52.06% were newly diagnosed cases. In contrast, in NFHS-4, a total of 83,997 previously diagnosed hypertension cases and 69,387 new hypertension cases were detected on screening. The prevalence of hypertension (cumulative previously diagnosed and new cases) among individuals aged 15 to 49 years was 20.4% (20.2%, 20.6%; *n *= 153,384), out of which 41.65% were newly diagnosed cases.

**Table 1:** Sociodemographic, anthropometric, and lifestyle characteristics of the study population (NFHS-5). NFHS-4, National Family Health Survey Fourth Series; NFHS-5, National Family Health Survey Fifth Series; BMI, body mass index

Variables	NFHS-4 (2015-2016)		NFHS-5 (2019-2021)	
	Previously diagnosed cases (*n* = 83,997)	New cases (*n* = 69,387)	Previously diagnosed cases (*n* = 82,718)	New cases (*n* = 89,814)
Sex				
Male	47.09 (45.6, 48.59)	52.91 (51.41, 54.40)	43.59 (41.64, 45.57)	56.41 (54.43, 58.36)
Female	60.36 (59.61, 61.11)	39.64 (38.89, 40.39)	52.80 (52.02, 53.57)	47.20 (46.43, 47.98)
Age group (in years)				
Adolescent (15-19)	64.82 (63.24, 66.37)	35.18 (33.63, 36.76)	59.47 (57.87, 61.06)	40.53 (38.94, 42.13)
Young (20-39)	60.47 (59.61, 61.32)	39.53 (38.68, 40.39)	52.86 (51.89, 53.83)	47.14 (46.17, 48.11)
Middle-aged (≥40 to 49)	53.72 (52.88, 54.57)	46.28 (45.43, 47.12)	47.16 (46.38, 47.95)	52.84 (52.05, 53.62)
Anemia (NHFS-4, *n* = 138,121; NFHS-5, *n* = 138,006)				
Severe	65.82 (61.53, 69.88)	34.18 (30.12, 38.47)	49.08 (46.30, 51.85)	50.92 (48.15, 53.71)
Moderate	59.52 (57.99, 61.03)	40.48 (38.97, 42.01)	41.71 (40.58, 42.82)	58.30 (57.18, 59.42)
Mild	56.65 (55.61, 57.68)	43.35 (42.32, 44.39)	39.81 (38.71, 40.92)	60.19 (59.08, 61.29)
Not anemic	48.44 (47.54. 49.34)	51.56 (50.66, 52.46)	35.87 (35.03, 36.71)	64.13 (63.29, 64.97)
BMI (NFHS-4, *n* = 139,521; NFHS-5, *n* = 144,726)				
Underweight	53.85 (52.55, 55.14)	46.15 (44.86, 47.45)	39.63 (38.29, 40.99)	60.37 (59.01, 61.71)
Normal weight	52.02 (51.03, 53.01)	47.98 (46.99, 48.97)	37.39 (36.47, 38.32)	62.61 (61.68, 63.53)
Overweight/Obese	53.14 (52.16, 54.12)	46.86 (45.88, 47.84)	41.36 (40.51, 42.21)	58.64 (57.79, 59.49)
Waist-to-hip ratio (NFHS-4, *n* = 144,392)				
<=0.9 for males and <=0.8 for females	-	-	36.51 (35.28, 37.76)	63.49 (62.24, 64.72)
Cutoffs > 0.9 for males and >0.8 for females	-	-	39.58 (38.81, 40.38)	60.42 (59.62, 61.20)
Marital status				
Never in union	61.93 (60.53, 63.31)	38.07 (36.69, 39.47)	58.31 (56.89, 59.68)	41.70 (40.32, 43.11)
Currently married	57.94 (57.18, 58.71)	42.06 (41.30, 42.82)	50.20 (49.42, 50.98)	49.81 (49.02, 50.58)
Widowed/Divorced/Separated	54.54 (52.73, 56.33)	45.46 (43.67, 47.27)	45.71 (44.18, 47.24)	54.29 (52.76, 55.82)
Education				
No education/Preprimary education	52.39 (51.47, 53.31)	47.61 (46.70, 48.53)	47.38 (46.48, 48.27)	52.62 (51.73, 53.52)
Primary	53.98 (52.78, 55.18)	46.02 (44.82, 47.22)	46.75 (45.51, 47.99)	53.25 (52.01, 54.49)
Secondary education	60.69 (59.77, 61.61)	39.31 (38.39, 40.23)	51.65 (50.71, 52.61)	48.35 (47.39, 49.31)
Higher education	66.65 (65.11, 68.16)	33.35 (31.84, 34.89)	61.19 (59.58, 62.78)	38.81 (37.22, 40.42)
Residence				
Urban	63.36 (62.01, 64.68)	36.64 (35.32, 37.99)	56.75 (55.29, 58.21)	43.25 (41.81, 44.71)
Rural	54.72 (53.90, 55.53)	45.28 (44.47, 46.10)	48.32 (47.43, 49.21)	51.68 (50.79, 52.57)
Cast/Tribe (NFHS-4, *n* = 144,521; NFHS-5, *n* = 161,782)				
Scheduled caste	59.06 (57.61, 60.49)	40.94 (39.51, 42.39)	51.05 (49.77, 52.34)	48.95 (47.66, 50.23)
Scheduled tribe	44.37 (42.68, 46.08)	55.63 (53.92, 57.32)	40.02 (38.52, 41.54)	59.98 (58.46, 61.48)
Other backward caste	60.24 (59.21, 61.27)	39.76 (38.73, 40.79)	53.52 (52.60, 54.43)	46.48 (45.57, 47.41)
Others	59.93 (58.83, 61.03)	40.07 (38.97, 41.17)	54.58 (53.16, 56.00)	45.42 (44.00, 46.84)
Religion				
Hindu	58.38 (57.54, 59.21)	41.62 (40.79, 42.46)	50.78 (49.96, 51.60)	49.22 (48.41, 50.04)
Muslim	59.16 (57.74, 60.55)	40.84 (39.45, 42.26)	52.33 (50.22, 54.44)	47.67 (45.56, 49.78)
Others	56.26 (54.41, 58.1)	43.74 (41.90, 45.59)	57.88 (55.70, 60.02)	42.12 (39.98, 44.31)
Wealth index				
Poorest	46.82 (45.64, 48.00)	53.18 (52.00, 54.36)	46.91 (45.40, 48.41)	53.11 (51.59, 54.61)
Poorer	53.83 (52.69, 54.97)	46.17 (45.03, 47.31)	46.53 (45.33, 47.73)	53.47 (52.27, 54.67)
Middle	58.37 (57.22, 59.51)	41.63 (40.49, 42.78)	48.08 (46.95, 49.22)	51.92 (50.78, 53.05)
Richer	60.57 (59.35, 61.78)	39.43 (38.22, 40.65)	51.52 (50.33, 52.71)	48.48 (47.31, 49.67)
Richest	64.64 (63.28, 65.97)	35.36 (34.03, 36.72)	61.18 (59.76, 62.57)	38.82 (37.43, 40.24)
Type of healthcare facility accessed (NFHS-4, *n* = 44,611; NFHS-5, *n* = 58,407)				
Public facility	60.33 (59.06, 61.58)	39.67 (38.42, 40.94)	48.69 (47.55, 49.83)	51.31 (50.17, 52.45)
Private facility	61.46 (60.15, 62.75)	38.54 (37.25, 39.85)	54.84 (52.41, 56.25)	45.16 (43.75, 46.59)
NGO/Other	54.82 (48.97, 60.54)	45.18 (39.46, 51.03)	48.17 (42.07, 54.32)	51.83 (45.68, 57.93)
Health insurance coverage				
No	57.67 (56.89, 58.44)	42.33 (41.56, 43.11)	52.87 (51.95, 53.79)	47.13 (46.21, 48.05)
Yes	60.52 (59.18, 61.85)	39.48 (38.15, 40.82)	48.09 (47.11, 49.08)	51.91 (50.92, 52.89)
Comorbidities				
Diabetes (NFHS-4, *n* = 150,618; NFHS-5, *n* = 170,399)	64.54 (62.02, 66.97)	35.46 (33.03, 37.98)	60.18 (58.16, 62.17)	39.82 (37.83, 41.84)
Heart disease (NFHS-4,*n* = 152,395; NFHS-5, *n* = 171,603)	69.95 (67.71, 72.09)	30.05 (27.91, 32.29)	63.33 (59.90, 66.63)	36.67 (33.37, 40.10)
Any tobacco use				
No	60.41 (59.65, 61.18)	39.59 (38.82, 40.35)	52.62 (51.81, 53.42)	47.38 (46.58, 48.19)
Yes	45.23 (44, 46.47)	54.77 (53.53, 56)	41.00 (39.43, 42.59)	59.01 (57.41, 60.57)
Alcohol usage current				
No	59.46 (58.72, 60.19)	40.54 (39.81, 41.28)	52.17 (51.36, 52.97)	47.83 (47.03, 48.64)
Yes	43.23 (41.09, 45.4)	56.77 (54.6, 58.91)	37.31 (34.86, 39.81)	62.70 (60.20, 65.14)
Fried food				
Less frequent	59.12 (58.25, 59.98)	40.88 (40.02, 41,75)	52.01 (51.16, 52.86)	47.99 (47.14, 48.84)
More frequent	57.47 (56.57, 58.35)	42.53 (41.65, 43.43)	50.76 (49.70, 51.83)	49.24 (48.17, 50.30)
Aerated drinks				
Less frequent	56.74 (56, 57.48)	43.26 (42.52, 44)	50.78 (49.97, 51.58)	49.22 (48.42, 50.03)
More frequent	62.71 (61.48, 63.92)	37.31 (36.08, 38.52)	54.72 (53.27, 56.16)	45.28 (43.84, 46.73)
Region				
North	65.31 (63.82, 66.75)	34.71 (33.25, 36.18)	59.28 (58.21, 60.35)	40.72 (39.65, 41.79)
Central	52.51 (51.41, 53.6)	47.49 (46.4, 48.59)	57.19 (56.03, 58.33)	42.81 (41.67, 43.97)
East	55.11 (53.68, 56.53)	44.90 (43.47, 46.32)	50.22 (47.99, 52.46)	49.78 (47.54, 52.01)
Northeast	46.28 (44.8, 47.77)	53.72 (52.23, 55.2)	42.34 (40.65, 44.06)	57.66 (55.94, 59.35)
West	50.67 (48.74, 52.59)	49.33 (47.41, 51.26)	45.71 (43.04, 48.41)	54.29 (51.59, 56.96)
South	65.83 (64.09, 67.53)	34.17 (32.47, 35.91)	47.55 (45.95, 49.14)	52.45 (50.86, 54.05)

In NFHS-5, among the hypertension cases, a majority of males (56.41%), middle-aged (52.84%), rural residents (51.68%), tobacco users (59%), and alcohol users (62.7%) were new cases that were previously undiagnosed. A majority of patients in the lower wealth quintiles (53.1%) and having lower educational status (53.25 % with just primary education) were newly diagnosed cases on screening compared to those from higher wealth quintiles and higher educational status, respectively. The region-wide distribution of the old and new cases was fairly similar across all categories except the northeastern region where new cases were the highest (57.66%).

The sociodemographic, anthropometric, and lifestyle characteristics associated with hypertension awareness status are reported in Table 2. Middle-aged individuals (adjusted odds ratios [aOR] = 2.51 and *P* < 0.001) and overweight or obese participants (aOR = 1.98 and *P *< 0.001) were found to be more aware of their hypertension status. Although the residents of rural areas, individuals possessing health insurance, and those not drinking alcohol have higher odds of being aware of their hypertension status; however, these associations were not statistically significant.

**Table 2:** Hypertension status awareness (previously told by a professional) distribution across sociodemographic, anthropometry, and lifestyle factors in NFHS-5. OBC, other backward class; Ref, reference; NFHS-5, National Family Health Survey Fifth Series; BMI, body mass index; NGO, nongovernmental organization; BP, blood pressure

Variables	Previously not told they had high BP (*n* = 34,972)	Previously told they had high BP on two or more occasions (*n* = 47,746)	Unadjusted odds	*P*-value	Adjusted odds	*P*-value
Sex						
Male	69.11 (66.66, 71.45)	30.89 (28.55, 33.34)	Ref		Ref	
Female	44.73 (43.69, 54.77)	55.27 (54.23, 56.31)	2.76 (2.47, 3.08)	<0.001	1.31 (0.95, 1.81)	0.09
Age group (in years)						
Adolescent (15-19)	81.40 (80.17, 82.56)	18.61 (17.44, 19.83)	Ref		Ref	
Young (20-39)	50.56 (49.29, 51.84)	49.44 (48.16, 50.71)	4.27 (3.96, 4.61)		1.30 (0.98, 1.74)	
Middle-aged (≥40 to 49)	31.01 (29.78, 32.27)	68.99 (67.73, 70.22)	9.73 (8.94, 10.58)	<0.001	2.51 (1.80, 3.51)	<0.001
Anemia (*n* = 51,680)						
Severe	11.18 (9.04, 13.76)	88.82 (86.24, 90.96)	Ref		-	
Moderate	12.30 (11.48, 13.16)	87.71 (86.84, 88.52)	0.89 (0.70, 1.14)			
Mild	12.28 (11.44, 13.19)	87.72 (86.81, 88.56)	0.89 (0.70, 1.15)			
Not anemic	11.53 (10.88, 12.21)	88.47 (87.79, 98.12)	0.96 (0.75, 1.23)	0.29		
BMI (*n* = 55,169)						
Underweight	23.82 (22.31, 25.4)	76.18 (74.61, 77.69)	Ref		Ref	
Normal weight	16.06 (15.31, 16.84)	83.94 (83.16, 84.70)	1.63 (1.48, 1.79)		1.24 (1.03, 1.50)	
Overweight/Obese	8.15 (7.60, 8.73)	91.85 (91.27, 92.40)	3.52 (3.12, 3.93)	<0.001	1.98 (1.57, 2.50)	<0.001
Waist-to-hip ratio (*n* = 54,874)						
<=0.9 for males and <=0.8 for females	18.02 (16.83, 19.28)	81.98 (80.72, 83.17)	Ref		Ref	
Cutoffs > 0.9 for males and >0.8 for females	12.18 (11.67, 12.71)	87.82 (87.29, 88.33)	1.58 (1.45, 1.73)	<0.001	1.03 (0.87, 1.22)	0.65
Marital status						
Never in union	80.83 (79.67, 81.94)	19.17 (18.06, 20.33)	Ref		Ref	
Currently married	39.31 (38.26, 40.35)	60.71 (59.65, 61.74)	6.50 (6.10, 6.94)		2.06 (1.59, 2.66)	
Widowed/Divorced/Separated	32.74 (30.47, 35.09)	67.26 (64.91, 69.53)	8.66 (7.69, 9.74)	<0.001	2.35 (1.57, 3.53)	<0.001
Education						
No education/Preprimary education	36.76 (35.48, 38.05)	63.24 (61.95, 64.52)	Ref		Ref	
Primary	38.24 (36.59, 39.92)	61.76 (60.08, 63.41)	0.93 (0.86, 1.01)		0.93 (0.74, 1.17)	
Secondary education	49.63 (48.45, 50.81)	50.37 (49.19, 51.55)	0.58 (0.55, 0.62)		0.98 (0.82, 1.17)	
Higher education	62.06 (59.88, 64.21)	37.94 (54.80, 40.12)	0.35 (0.32, 0.39)	<0.001	1.18 (0.91, 1.53)	0.34
Residence						
Urban	56.62 (54.66, 58.55)	43.38 (41.45, 45.34)	Ref		Ref	
Rural	41.48 (40.51, 42.46)	58.52 (57.54, 59.49)	1.84 (1.68, 2.01)	<0.001	0.91 (0.75, 1.11)	0.40
Cast/Tribe (*n* = 78,206)						
Scheduled caste	43.93 (42.32, 45.55)	56.07 (54.45, 57.68)	1.28 (1.16, 1.41)		1.11 (0.88, 1.39)	
Scheduled tribe	51.28 (48.82, 53.76)	48.72 (46.24, 51.22)	0.95 (0.84, 1.08)		1.06 (0.84, 1.41)	
OBC	46.62 (45.25, 47.99)	52.38 (52.01, 54.75)	1.15 (1.05, 1.25)		1.10 (0.91, 1.33)	
Others	50.15 (48.16, 52.15)	49.85 (57.85, 51.84)	Ref	<0.001	Ref	0.75
Religion						
Hindu	46.29 (45.14, 47.44)	53.71 (52.56, 54.86)	Ref		Ref	
Muslim	53.76 (51.50, 56.01)	46.24 (43.99, 48.51)	0.74 (0.67, 0.81)		0.95 (0.77, 1.17)	
Others	49.41 (45.79, 53.01)	50.61 (46.99, 54.21)	0.88 (0.76, 1.01)	<0.001	0.94 (0.69, 1.27)	0.83
Wealth index						
Poorest	46.76 (45.14, 48.39)	53.24 (51.61, 54.86)	Ref		Ref	
Poorer	42.85 (41.33, 44.38)	57.15 (55.62, 58.67)	1.17 (1.08, 1.26)		1.29 (1.04, 1.60)	
Middle	41.24 (39.68, 42.81)	58.76 (57.19, 60.32)	1.25 (1.14, 1.36)		1.32 (1.05, 1.66)	
Richer	45.83 (44.11, 47.56)	54.17 (52.44, 55.90)	1.03 (0.94, 1.13)		1.38 (1.04, 1.82)	
Richest	56.78 (54.74, 58.80)	43.22 (41.21, 45.26)	0.67 (0.60, 0.74)	<0.001	1.32 (0.96, 1.83)	0.09
Type of healthcare facility accessed (*n* = 28,715)						
Public facility	35.94 (34.61, 37.32)	64.06 (62.71, 65.49)	Ref		Ref	
Private facility	41.63 (39.56, 43.73)	58.37 (56.27, 60.44)	0.78 (0.71, 0.86)		1.05 (0.91, 1.22)	
NGO/Other	33.95 (26.27, 42.55)	66.06 (57.45, 73.73)	1.09 (0.75, 1.57)	<0.001	2.26 (1.11, 4.58)	0.06
Health insurance coverage						
No	49.43 (48.24, 50.62)	50.57 (49.38, 51.76)	Ref		Ref	
Yes	43.17 (41.64, 44.71)	56.83 (55.29, 58.36)	1.28 (1.20, 1.37)	<0.001	0.88 (0.76, 1.02)	0.11
Comorbidities						
Diabetes (*n* = 81,845)						
No	49.17 (48.08, 50.27)	50.83 (49.73, 51.92)	Ref		Ref	
Yes	17.04 (14.89, 19.44)	82.96 (80.56, 85.11)	4.7 (4.01, 5.51)	<0.001	1.34 (0.93, 1.93)	0.11
Heart disease (*n* = 82,372)						
No	48.12 (47.04, 49.21)	51.88 (50.80, 52.96)	Ref		Ref	
Yes	20.59 (16.88, 24.87)	79.41 (75.13, 83.12)	3.57 (2.81, 4.55)	<0.001	1.25 (0.69, 2.24)	0.44
Any tobacco use						
No	47.55 (46.46, 48.65)	52.45 (51.35, 53.54)	1.08 (0.98, 1.19)	0.10	-	
Yes	49.53 (47.07, 51.99)	50.47 (48.01, 52.93)	Ref			
Alcohol usage currently						
No	47.36 (46.28, 48.44)	52.64 (51.56, 53.72)	Ref		Ref	
Yes	57.60 (53.38, 61.72)	42.40 (38.28, 46.62)	0.66 (0.55, 0.78)	<0.001	1.02 (0.65, 1.60)	0.90
Fried food						
Less frequent	47.40 (46.20, 48.61)	52.60 (51.39, 53.80)	Ref		-	
More frequent	48.12 (46.74, 49.50)	51.88 (50.50, 53.26)	0.97 (0.91, 1.02)	0.317		
Aerated drinks						
Less frequent	45.68 (44.66, 46.71)	54.32 (53.32, 55.34)	Ref		Ref	
More frequent	56.64 (54.49, 58.76)	43.36 (41.24, 45.51)	0.64 (0.59, 0.69)	<0.001	0.79 (0.66, 0.94)	0.009
Region						
North	44.10 (42.57, 45.63)	55.91 (54.37, 57.43)	Ref		Ref	
Central	49.69 (47.86, 51.53)	50.31 (48.47, 52.14)	0.79 (0.72, 0.87)		0.97 (0.77, 1.21)	
East	38.17 (36.37, 40.00)	61.83 (60.00, 63.63)	1.27 (1.15, 1.41)		1.01 (0.79, 1.28)	
Northeast	37.01 (34.67, 39.41)	62.99 (60.59, 65.33)	1.34 (1.19, 1.51)		0.53 (0.40, 0.70)	
West	65.71 (62.11, 69.13)	34.31 (30.87, 37.91)	0.41 (0.34, 0.48)		0.40 (0.30, 0.53)	
South	48.59 (46.14, 51.04)	51.41 (48.96, 53.86)	0.83 (0.74, 0.93)	<0.001	0.77 (0.64, 0.98)	<0.001

In NFHS-5, 40.7% (39.8%, 41.6%) of the previously diagnosed cases were initiated on BP-lowering medications. However, in NFHS-4, about 32.6% (31.8%, 33.6%) of the previously diagnosed cases were on BP-lowering medications. The determinants of positive treatment-seeking behavior considered in patients on BP-lowering medications are reported in Table 3. A significantly higher proportion of middle-aged (53.47%), those who were separated from their partners (53.49%), and those who belonged to the richest wealth quintiles (45.08%) were on BP-lowering medications. Contrary to this, females compared to males (aOR = 0.72 and *P* = 0.007), residents of rural areas (aOR = 0.82 and *P* = 0.004), and those belonging to the socially disadvantaged groups - scheduled caste [SC]/scheduled caste [ST]/another backward caste [OBC] - were less likely to be on BP-lowering medications.

**Table 3:** Treatment-seeking behavior in NFHS-5 among patients aware of being hypertensive (N = 52,920). OBC, other backward class; Ref, reference; NFHS-5, National Family Health Survey Fifth Series; BMI, body mass index; NGO, nongovernmental organization; BP, blood pressure; SC, scheduled caste; ST, scheduled tribe

Variables	Not taking medicine (*n* = 32,223)	Taking medicine (*n* = 20,697)	Unadjusted odds	*P*-value	Adjusted odds	*P*-value
Sex						
Male	50.98 (47.72, 54.24)	49.02 (45.76, 52.28)	Ref		Ref	
Female	59.95 (59.06, 60.84)	40.05 (39.16, 40.94)	0.69 (0.61, 0.79)	<0.001	0.72 (0.57, 0.91)	0.007
Age group (in years)						
Adolescent (15-19)	58.01 (55.36, 60.62)	41.99 (39.38, 44.64)	Ref		Ref	
Young (20-39)	69.80 (68.89, 70.79)	30.20 (29.21, 31.20)	0.59 (0.53, 0.66)		0.91 (0.70, 1.18)	
Middle-aged (≥40 to 49)	46.53 (45.42, 47.64)	53.47 (52.36, 54.58)	1.58 (1.42, 1.77)	<0.001	2.15 (1.64, 2.83)	<0.001
Anemia (*n* = 50,424)						
Severe	63.35 (59.51, 67.03)	36.65 (32.97, 40.49)	Ref		-	
Moderate	59.20 (57.83, 60.55)	40.80 (39.45, 42.17)	1.19 (1.01, 1.41)			
Mild	60.61 (59.18, 62.02)	39.39 (37.98, 40.82)	1.12 (0.94, 1.32)			
Not anemic	60.30 (59.12, 61.46)	39.70 (38.54, 40.88)	1.13 (0.96, 1.34)	0.12		
BMI (*n* = 52,778)						
Underweight	65.44 (63.61, 67.23)	34.56 (32.77, 36.4)	Ref		Ref	
Normal weight	64.15 (63.05, 65.25)	35.85 (34.75, 36.95)	1.05 (0.97, 1.15)		0.95 (0.82, 1.11)	
Overweight/obese	51.91 (50.73, 53.09)	48.09 (46.91, 49.27)	1.75 (1.60, 1.91)	<0.001	1.39 (1.18, 1.63)	<0.001
Waist-to-hip ratio (*n* = 52,755)						
<=0.9 for males and <=0.8 for females	63.01 (61.31, 64.67)	36.99 (35.33, 38.69)	Ref		Ref	
Cutoffs > 0.9 for males and >0.8 for females	58.51 (57.56, 59.45)	41.51 (40.55, 42.44)	1.21 (1.12, 1.29)	<0.001	1.01 (0.89, 1.15)	0.77
Marital status						
Never in union	59.95 (57.79, 62.08)	40.05 (37.92, 42.21)	Ref		Ref	
Currently married	60.09 (59.16, 61)	39.91 (39.00, 40.84)	0.99 (0.91, 1.08)		0.78 (0.63, 0.98)	
Widowed/Divorced/Separated	46.51 (43.98, 49.06)	53.49 (50.94, 56.02)	1.72 (1.51, 1.95)	<0.001	1.12 (0.85, 1.49)	<0.001
Education						
No education/Preprimary education	59.15 (57.80, 60.49)	40.85 (39.51, 42.20)	Ref		Ref	
Primary	56.29 (54.54, 58.03)	43.71 (41.97, 45.46)	1.12 (1.03, 1.22)		1.09 (0.94, 1.26)	
Secondary education	59.06 (57.95, 60.17)	40.94 (39.83, 42.05)	1.00 (0.94, 1.06)		1.02 (0.91, 1.15)	
Higher education	63.33 (61.27, 65.35)	36.67 (34.65, 38.73)	0.83 (0.75, 0.92)	<0.001	0.80 (0.66, 0.98)	0.02
Residence						
Urban	53.49 (51.68, 55.28)	46.51 (44.72, 48.32)	Ref		Ref	
Rural	62.21 (61.21, 63.22)	37.79 (36.81, 38.79)	0.69 (0.64, 0.75)	<0.001	0.82 (0.72, 0.94)	0.004
Cast/Tribe (*n* = 50,035)						
SC	63.61 (62.01, 65.17)	36.39 (34.83, 37.99)	0.74 (0.68, 0.81)		0.79 (0.68, 0.92)	
ST	57.27 (54.78, 59.72)	42.73 (40.28, 45.22)	0.97 (0.86, 1.09)		1.02 (0.84, 1.24)	
OBC	60.94 (59.72, 62.17)	39.06 (37.83, 40.30)	0.83 (0.77, 0.91)	<0.001	0.90 (0.80, 1.02)	0.01
Non-SC/non-ST/non-OBC	56.71 (55.14, 58.26)	43.29 (41.74, 44.86)	Ref		Ref	
Religion						
Hindu	60.18 (59.21, 61.15)	39.82 (38.85, 40.8)	Ref		Ref	
Muslim	53.69 (51.35, 56.01)	46.31 (43.99, 48.65)	1.30 (1.18, 1.43)		1.12 (0.96, 1.30)	
Others	60.66 (57.94, 63.31)	39.34 (36.69, 42.06)	0.98 (0.87, 1.10)	<0.001	1.01 (0.84, 1.21)	0.31
Wealth index						
Poorest	64.50 (62.74, 66.23)	35.51 (33.77, 37.26)	Ref		Ref	
Poorer	64.01 (62.52, 65.47)	35.99 (34.53, 37.48)	1.02 (0.93, 1.11)		1.03 (0.88, 1.20)	
Middle	59.13 (57.56, 60.67)	40.87 (39.33, 42.44)	1.25 (1.14, 1.37)		1.23 (1.04, 1.46)	
Richer	56.18 (54.62, 57.75)	43.82 (42.25, 45.41)	1.41 (1.28, 1.56)		1.21 (1.02, 1.44)	
Richest	54.92 (53.25, 56.57)	45.08 (43.43, 46.75)	1.49 (1.34, 1.64)	<0.001	1.14 (0.93, 1.39)	0.03
Type of healthcare facility accessed (*n* = 20,663)						
Public facility	60.16 (58.64, 61.67)	39.84 (38.33, 41.36)	Ref		Ref	
Private facility	58.63 (56.89, 60.34)	41.37 (39.66, 43.11)	1.06 (0.97, 1.16)		0.98 (0.89, 1.07)	
NGO/Other	47.00 (38.11, 56.09)	53.00 (43.91, 61.91)	1.70 (1.18, 2.45)	0.008	1.63 (1.10, 2.42)	0.04
Health insurance coverage						
Yes	52.43 (51.07, 53.79)	47.57 (46.21, 48.93)	1.49 (1.40, 1.59)	<0.001	1.55 (1.40, 1.71)	<0.001
No	62.21 (61.18, 63.23)	37.79 (36.77, 38.82)	Ref		Ref	
Comorbidities						
Diabetes (n = 52,214)						
No	61.15 (60.23, 62.05)	38.85 (37.95, 39.77)	Ref		Ref	
Yes	32.71 (30.29, 35.23)	67.29 (64.77, 69.71)	3.23 (2.88, 3.63)	<0.001	2.22 (1.85, 2.65)	<0.001
Heart disease (*n* = 52,648)						
No	59.69 (58.79, 60.59)	40.31 (39.41, 41.21)	Ref		Ref	
Yes	42.01 (37.54, 46.61)	57.99 (53.39, 62.46)	2.04 (1.69, 2.46)	<0.001	1.44 (1.06, 1.97)	0.01
Usage of any tobacco						
No	59.71 (58.79, 60.62)	40.29 (39.38, 41.21)	0.79 (0.71, 0.88)	<0.001	0.85 (0.70, 1.03)	0.10
Yes	54.11 (51.53, 56.70)	45.89 (43.30, 48.50)	Ref		Ref	
Alcohol usage currently						
No	59.57 (58.67, 60.47)	40.43 (39.53, 41.33)	Ref		Ref	
Yes	49.31 (44.67, 53.93)	50.70 (46.07, 55.33)	1.51 (1.25, 1.82)	<0.001	1.08 (0.79, 1.47)	0.61
Fried food						
Less frequently	59.44 (58.38, 60.51)	40.56 (39.51, 41.62)	Ref		-	
More frequently	59.07 (57.84, 60.28)	40.93 (39.72, 42.16)	1.01 (0.95, 1.07)	0.59		
Aerated drinks						
Less frequently	59.71 (58.77, 60.64)	40.29 (39.36, 41.23)	Ref		Ref	
More frequently	57.00 (55.11, 58.87)	43.00 (41.13, 44.89)	1.11 (1.03, 1.20)	0.006	1.18 (1.03, 1.34)	0.01
Region						
North	66.34 (64.94, 67.72)	33.66 (32.28, 35.06)	Ref		Ref	
Central	73.26 (71.86, 74.62)	26.74 (25.38, 28.14)	0.71 (0.65, 0.79)		0.71 (0.60, 0.84)	
East	60.47 (58.55, 62.36)	39.53 (37.64, 41.45)	1.28 (1.16, 1.42)		1.35 (1.12, 1.62)	
Northeast	47.16 (44.87, 49.47)	52.84 (50.53, 55.13)	2.21 (1.97, 2.46)		2.23 (1.79, 2.77)	
West	40.28 (37.56, 43.06)	59.72 (56.94, 62.44)	2.92 (2.56, 3.33)		2.62 (2.02, 3.39)	
South	46.09 (43.61, 48.60)	53.91 (51.42, 56.42)	2.30 (2.04, 2.59)	<0.001	2.39 (2.01, 2.85)	<0.001

In NFHS-5, controlled BP was observed in 73.7% (72.7%, 74.7%) of patients on BP-lowering medication, while 80.8% (80.0%, 81.6%) of patients taking hypertension medication had controlled BP in NFHS-4. Among patients on antihypertensive medication, those reporting consuming alcohol, tobacco smoking, frequent consumption of fried food, presence of diabetes comorbidity, and lacking higher education had significantly lower odds of BP control compared to their counterparts. Factors such as increasing age (aOR = 0.49 and *P *< 0.001), higher BMI (aOR = 0.51, *P *< 0.001 for obese/overweight), and greater waist-to-hip ratio (aOR = 0.78 and *P *= 0.047) were also associated with poor control of hypertension despite medication therapy. Only females (aOR = 1.7 and *P *= 0.003) and individuals with higher education levels (aOR = 1.5 and *P *< 0.004) when on drug treatment were associated with higher odds of achieving control over their BP levels (Table 4).

**Table 4:** Predictors associated with controlled hypertension in patients on antihypertensive treatment (N = 20,697). OBC, other backward class; Ref, reference; BMI, body mass index; NGO, nongovernmental organization; BP, blood pressure; SC, scheduled caste; ST, scheduled tribe

Variables	Uncontrolled hypertension (*n* = 5,580)	Controlled hypertension (*n* = 15,117)	Unadjusted odds	*P*-value	Adjusted odds	*P*-value
Sex						
Male	34.28 (29.89, 38.96)	65.72 (61.04, 70.11)	Ref		Ref	
Female	25.47 (24.53, 26.44)	74.53 (73.56, 75.47)	1.52 (1.24, 1.87)	<0.001	1.70 (1.21, 2.41)	0.003
Age group (in years)						
Adolescent (15-19)	8.21 (6.12, 10.94)	91.79 (89.06, 93.88)	Ref		Ref	
Young (20-39)	19.07 (17.67, 20.56)	80.93 (79.44, 82.33)	0.37 (0.27, 0.52)		0.88 (0.49, 1.56)	
Middle-aged (≥40 to 49)	33.04 (31.75, 34.36)	66.96 (65.64, 68.25)	0.18 (0.13, 0.24)	<0.001	0.49 (0.27, 0.89)	<0.001
Anemia (*n* = 19,321)						
Severe	20.87 (16.22, 26.42)	79.13 (73.58, 83.78)	Ref		-	
Moderate	20.49 (18.91, 22.18)	79.51 (77.82, 81.11)	1.02 (0.74, 1.41)			
Mild	21.71 (20.01, 23.48)	78.30 (76.52, 79.99)	0.95 (0.68, 1.31)			
Not anemic	23.39 (21.95, 24.89)	76.61 (75.11, 78.05)	0.86 (0.63, 1.18)	0.06		
BMI (*n* = 20,635)						
Underweight	11.36 (9.60, 13.39)	88.64 (86.61, 90.39)	Ref		Ref	
Normal weight	23.36 (22.04, 24.73)	76.64 (75.27, 77.96)	0.42 (0.34, 0.51)		0.63 (0.44, 0.89)	
Overweight/obese	31.58 (30.14, 33.06)	68.42 (66.94, 69.86)	0.22 (0.23, 0.34)	<0.001	0.51 (0.35, 0.72)	<0.001
Waist-to-hip ratio (*n* = 20,630)						
<=0.9 for males and <=0.8 for females	19.23 (16.98, 21.70)	80.77 (78.3, 83.02)	Ref		Ref	
Cutoffs > 0.9 for males and >0.8 for females	27.55 (26.5, 28.62)	72.45 (71.38, 73.5)	0.62 (0.53, 0.73)	<0.001	0.78 (0.61, 0.99)	0.04
Marital status						
Never in union	13.01 (10.95, 15.42)	86.99 (84.6, 89.05)	Ref		Ref	
Currently married	27.03 (25.97, 28.12)	72.97 (71.88, 74.03)	0.40 (0.33, 0.49)		0.78 (0.53, 1.14)	
Widowed/Divorced/Separated	33.67 (30.41, 37.12)	66.33 (62.9, 69.59)	0.29 (0.23, 0.37)	<0.001	0.62 (0.39, 1.00)	0.11
Education						
No education/Preprimary education	29.97 (28.26, 31.73)	70.03 (68.27, 71.74)	Ref		Ref	
Primary	28.53 (26.32, 30.87)	71.74 (69.13, 73.70)	1.07 (0.93, 1.22)		1.16 (0.92, 1.46)	
Secondary education	24.38 (23.02, 25.79)	75.62 (74.21, 76.98)	1.33 (1.19, 1.47)		1.36 (1.13, 1.65)	
Higher education	22.44 (19.62, 25.53)	77.56 (74.47, 80.38)	1.48 (1.22, 1.78)	<0.001	1.58 (1.15, 2.15)	0.004
Residence						
Urban	28.12 (26.31, 30.01)	71.88 (69.99, 73.69)	Ref		Ref	
Rural	25.11 (24.03, 26.23)	74.89 (73.77, 75.97)	1.16 (1.04, 1.30)	0.005	0.96 (0.80, 1.14)	0.66
Cast/Tribe (*n* = 19,162)						
SC	25.43 (23.52, 27.43)	74.57 (72.57, 76.48)	1.02 (0.89, 1.17)		-	
ST	23.41 (20.74, 26.31)	76.59 (73.69, 79.26)	1.14 (0.95, 1.36)			
OBC	25.36 (23.93, 26.85)	74.64 (73.15, 76.07)	1.02 (0.91, 1.16)			
Non-SC/non-ST/non-OBC	25.89 (24.08, 27.79)	74.11 (72.21, 75.92)	Ref	0.53		
Religion						
Hindu	25.24 (24.19, 26.32)	74.76 (73.68, 75.81)	Ref		Ref	
Muslim	29.95 (27.36, 32.68)	70.05 (67.32, 72.64)	0.78 (0.68, 0.90)		0.81 (0.65, 1.00)	
Others	29.30 (26.03, 32.80)	70.71 (67.21, 73.97)	0.81 (0.68, 0.96)	<0.001	0.87 (0.65, 1.15)	0.66
Wealth index						
Poorest	22.25 (20.05, 24.61)	77.75 (75.39, 79.95)	Ref		Ref	
Poorer	23.93 (21.93, 26.05)	76.07 (73.95, 78.07)	0.91 (0.77, 1.07)		0.94 (0.70, 1.26)	
Middle	26.77 (24.87, 28.76)	73.23 (71.24, 75.13)	0.78 (0.66, 0.92)		0.81 (0.60, 1.09)	
Richer	27.40 (25.58, 29.29)	72.69 (70.71, 74.42)	0.75 (0.64, 0.88)		0.83 (0.60, 1.15)	
Richest	28.51 (26.49, 30.61)	71.49 (69.39, 73.51)	0.71 (0.61, 0.84)	<0.001	0.66 (0.45, 0.96)	0.15
Type of healthcare facility accessed (*n* = 8,103)						
Public facility	26.39 (24.58, 28.28)	73.61 (71.72, 75.42)	Ref		-	
Private facility	26.52 (24.31, 28.86)	73.48 (71.14, 75.7)	0.99 (0.85, 1.14)			
NGO/Other	41.91 (28.39, 56.74)	58.11 (43.26, 71.61)	0.49 (0.27, 0.91)	0.07		
Health insurance coverage						
Yes	27.71 (26.19, 29.29)	72.29 (70.71, 73.81)	0.89 (0.81, 0.98)	0.02	1.10 (0.94, 1.28)	0.23
No	25.49 (24.31, 26.71)	74.51 (73.29, 75.69)	Ref		Ref	
Comorbidities						
Diabetes (*n* = 20,443)						
No	25.28 (24.27, 26.32)	74.72 (73.68, 75.73)	Ref		Ref	
Yes	34.22 (31.25, 37.33)	65.78 (62.67, 68.75)	0.65 (0.56, 0.75)	<0.001	0.79 (0.63, 0.99)	0.04
Heart disease (*n* = 20,583)						
No	26.21 (25.23, 27.22)	73.79 (72.78, 74.77)	Ref		-	
Yes	26.43 (21.49, 32.04)	73.57 (67.96, 78.51)	0.98 (0.75, 1.30)	0.93		
Usage of any tobacco						
No	25.81 (24.85, 26.81)	74.19 (73.20, 75.15)	1.29 (1.07, 1.55)		0.85 (0.63, 1.15)	0.31
Yes	31.05 (27.33, 35.02)	68.95 (64.98, 72.67)	Ref	0.006	Ref	
Alcohol usage currently						
No	25.83 (24.88, 26.81)	74.17 (73.19, 75.12)	Ref		Ref	
Yes	38.21 (30.51, 46.53)	61.81 (53.47, 69.49)	0.56 (0.39, 0.79)	0.001	0.80 (0.48, 1.34)	0.41
Fried food						
Less frequently	25.56 (24.34, 26.81)	74.44 (73.19, 75.66)	Ref		-	
More frequently	27.20 (25.71, 28.75)	72.80 (71.25, 74.30)	0.92 (0.83, 1.01)	0.09		
Aerated drinks						
Less frequently	26.42 (25.37, 27.49)	73.58 (72.51, 74.63)	Ref		-	
More frequently	25.53 (23.41, 27.76)	74.47 (72.24, 76.59)	1.04 (0.92, 1.18)	0.45		
Region						
North	26.71 (24.98, 28.51)	73.29 (71.49, 75.02)	Ref		Ref	
Central	23.29 (21.41, 25.27)	76.71 (74.73, 78.59)	1.20 (1.04, 1.38)		0.90 (0.69, 1.17)	
East	25.99 (23.81, 28.31)	74.01 (71.70, 76.19)	1.03 (0.89, 1.20)		0.69 (0.53, 0.89)	
Northeast	33.71 (31.05, 36.47)	66.29 (63.53, 68.95)	0.71 (0.61, 0.83)		0.50 (0.37, 0.66)	
West	23.73 (20.38, 27.44)	76.27 (72.56, 79.62)	1.17 (0.94, 1.45)		0.96 (0.68, 1.35)	
South	27.85 (26.00, 29.77)	72.15 (20.23, 74.00)	0.94 (0.82, 1.07)	<0.001	1.08 (0.86, 1.34)	<0.001

Table 5 compares the proportion of hypertensive patients on treatment and achieving optimal BP control between various states and union territories of India stratified by their health index (performance) scores. Individuals in the majority of the Indian states have poor (<50%) treatment-seeking behavior due to the noninitiation of regular antihypertensive treatment despite awareness of their hypertension status. In empowered action group states such as Jharkhand and Uttar Pradesh, less than 25% of previously diagnosed hypertensives were on treatment at the time of the survey. However, a majority of states achieved BP control in 65% or more of hypertensive patients taking BP-lowering medications.

**Table 5:** Comparison of proportion of hypertensive patients on treatment and achieving optimal blood pressure in control in states and union territories of India stratified by health index scores. NFHS-4, National Family Health Survey Fourth Series; NFHS-5, National Family Health Survey Fifth Series; CI, confidence interval

States	Health index reference year (2019-2020)	Awareness of the hypertension status and on treatment, row% (95% CI) NFHS-5	On treatment with optimal control, row% (95% CI) NFHS-5	Awareness of the hypertension status and on treatment, row% (95% CI) NFHS-4	On treatment with optimal control, row% (95% CI) NFHS-4
High health index					
Kerala	82.21	59.96 (55.62, 64.14)	72.71 (68.02, 76.93)	49.59 (45.78, 53.41)	84.72 (80.34, 88.27)
Mizoram	75.77	44.57 (37.83, 51.52)	86.29 (78.69, 91.47)	24.13 (20.38, 28.33)	78.25 (70.61, 84.35)
Tamil Nadu	72.42	38.97 (34.64, 43.49)	71.06 (66.95, 74.86)	18.94 (17.18, 20.84)	80.24 (77.25, 82.93)
Tripura	70.16	51.64 (46.03, 57.20)	73.35 (66.68, 79.11)	46.12 (40.31, 52.05)	81.14 (75.63, 85.63)
Telangana	69.96	39.78 (39.03, 40.54)	72.96 (69.31, 73.81)	30.27 (26.22, 34.66)	79.09 (74.29, 83.21)
Andhra Pradesh	69.95	64.50 (59.82, 68.93)	76.46 (72.28, 80.19)	33.61 (29.17, 38.35)	79.58 (74.89, 83.59)
Maharashtra	69.14	61.94 (58.06, 65.67)	77.17 (72.55, 81.28)	43.53 (38.84, 48.34)	83.31 (79.56, 86.47)
Dadra and Nagar Haveli and Daman and Diu	66.21	53.98 (42.77, 64.81)	69.44 (51.31, 83.05)	62.54 (51.54, 72.39)	84.94 (73.18, 92.11)
Gujarat	63.59	53.63 (50.06, 57.17)	79.31 (75.09, 82.96)	45.57 (41.94, 49.24)	84.14 (79.94, 87.61)
Himachal Pradesh	63.17	39.46 (35.39, 43.68)	81.13 (76.06, 85.33)	35.25 (31.65, 39.02)	76.15 (70.92, 80.71)
Chandigarh	62.53	28.11 (17.91, 41.21)	93.73 (68.81, 99.02)	28.51 (20.53, 38.12)	72.73 (56.30, 84.67)
Punjab	58.08	28.11 (25.84, 39.48)	67.81 (63.76, 71.61)	32.56 (30.06, 35.16)	65.02 (60.77, 69.05)
Karnataka	57.93	55.58 (50.39, 60.66)	67.92 (63.34, 72.18)	43.61 (38.70, 48.66)	74.59 (70.29, 78.46)
Sikkim	55.53	44.22 (35.00, 53.86)	62.71 (46.98, 76.12)	32.72 (28.69, 37.02)	67.23 (60.41, 73.41)
Medium health index					
Goa	53.68	73.57 (62.36, 82.39)	44.76 (31.33, 58.99)	62.54 (51.54, 72.39)	79.50 (68.02, 87.61)
Lakshadweep	51.87	67.13 (51.68, 79.59)	72.42 (57.14, 83.79)	49.00 (38.43, 59.66)	72.99 (57.61, 84.31)
Puducherry	50.83	64.02 (50.36, 75.73)	77.41 (64.51, 86.61)	15.13 (9.21, 23.86)	75.14 (65.47, 82.81)
Chhattisgarh	50.71	38.26 (33.89, 42.81)	74.75 (69.62, 79.27)	40.57 (36.46, 44.82)	79.55 (75.00, 83.45)
Delhi	49.84	46.96 (42.78, 51.21)	68.04 (63.31, 72.43)	33.29 (26.22, 41.21)	82.44 (74.05, 88.54)
Haryana	49.26	29.44 (26.94, 32.08)	75.32 (71.59, 78.71)	23.45 (20.49, 26.71)	82.87 (79.00, 86.15)
Assam	47.74	54.81 (51.66, 57.91)	64.08 (60.41, 67.59)	41.83 (39.04, 44.67)	63.26 (59.71, 66.68)
Jharkhand	47.55	21.04 (18.35, 24.00)	81.02 (76.35, 84.95)	16.87 (14.99, 18.93)	84.74 (80.87, 87.95)
Jammu and Kashmir	46.99	48.66 (44.12, 53.23)	59.81 (55.27, 64.21)	42.77 (39.86, 45.73)	80.77 (77.98, 83.27)
Low health index					
A&N islands	44.74	71.11 (62.07, 70.71)	69.36 (58.81, 78.21)	57.87 (48.41, 66.81)	71.62 (63.06, 78.86)
Odisha	44.31	44.81 (41.24, 48.43)	76.33 (72.79, 79.54)	37.07 (33.9, 40.36)	82.92 (80.17, 85.36)
Uttarakhand	44.21	39.68 (34.76, 44.81)	67.27 (58.76, 74.79)	39.71 (36.16, 43.35)	78.97 (74.86 (82.56)
Meghalaya	43.05	72.39 (67.07, 77.15)	77.28 (71.81, 81.95)	52.21 (45.5, 58.85)	86.04 (82.16, 98.18)
Rajasthan	41.33	32.08 (29.16, 35.15)	81.70 (77.82, 85.02)	33.86 (31.48, 36.32)	78.25 (70.61, 84.35)
Madhya Pradesh	36.72	38.76 (35.57, 42.06)	76.74 (72.99, 80.11)	48.53 (38.84, 48.34)	84.78 (82.75 (86.61)
Manipur	34.26	36.09 (31.53, 40.91)	66.67 (57.82, 74.48)	20.29 (17.67, 23.18)	67.19 (61.18, 72.69)
Arunachal Pradesh	33.92	30.09 (26.98, 33.40)	68.33 (63.63, 72.69)	32.89 (29.30, 36.70)	64.08 (59.22, 68.66)
Bihar	31.00	29.34 (27.13, 31.65)	85.99 (83.46, 88.19)	42.19 (39.05, 45.41)	89.11 (86.98, 90.93)
Uttar Pradesh	30.57	23.34 (21.86, 24.89)	76.57 (73.97, 78.99)	26.47 (24.92, 28.08)	85.79 (83.97, 87.42)
Nagaland	27.01	31.59 (26.16, 37.59)	52.00 (43.79, 60.11)	26.47 (23.26, 29.96)	51.91 (45.28, 58.46)
West Bengal	Not available	62.43 (58.69, 66.04)	62.05 (57.79, 66.13)	42.87 (39.01, 46.81)	73.90 (69.92, 77.52)

**Figure 1:**
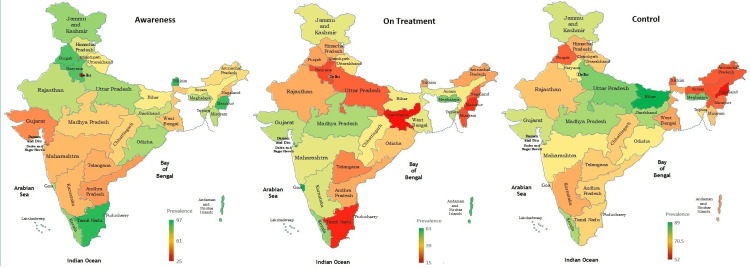
Prevalence of hypertension awareness, treatment, and control across states in India (NFHS-4, 2015-2016). Figure credits: All the authors of this study. NFHS-4, National Family Health Survey Fourth Series

**Figure 2:**
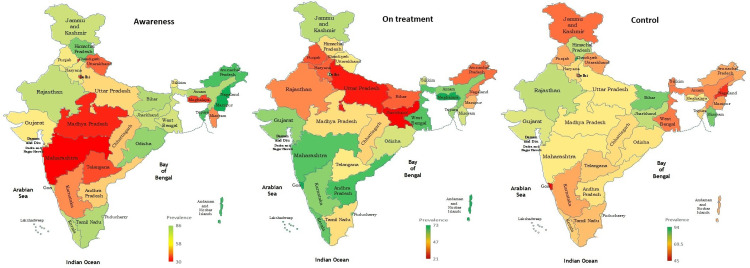
Prevalence of hypertension awareness, treatment, and control across states in India (NFHS-5, 2019-2021). Figure credits: All the authors of this study. NFHS-5, National Family Health Survey Fifth Series

Figures 1 and 2 display the change in state-wise prevalence of individuals with awareness of their hypertension status, on treatment, and with controlled BP. Furthermore, the correlation coefficient of the NITI Aayog health index score with a state-wise prevalence of aware individuals on treatment was 0.36 (*P *= 0.037) while correlation with the prevalence of individuals with controlled BP was 0.19 (*P *= 0.26).

## Discussion

This nationally representative survey in the age group of 15 to 49 years indicates a weak hypertension control cascade in India. The burden of hypertension increased slightly from 20.4% (NFHS-4) to 22.8% (NFHS-5), while the proportion of new cases detected on screening increased from 41.6% to 52.1%. The proportion of previously diagnosed cases on antihypertensive therapy increased from 32.6% to 40.7%. However, the proportion of cases of BP-lowering medication attaining controlled BP modestly reduced from 80.8% to 73.7%.

The overall prevalence of hypertension in India in the age group of 15-49 years as per NFHS-5 was 22.80%, an estimate similar to that reported in the District Level Health Survey and the Annual Health Survey (2012-2014), similar nationally representative surveys from India [15]. On comparing DHS surveys across countries, the prevalence of hypertension in India is higher than in Peru (19.77%) [16] and Nepal (19.99%) [17] but lower than in Bangladesh (27.5%) [18].

A majority (~58%) of existing hypertension cases in India are undiagnosed as per the current round of the NFHS, a finding almost identical to that in Bangladesh (2017-2018) [18]. The burden of undiagnosed cases was significantly higher in males, middle-aged, lower education level, poorer wealth quintiles, STs, and rural inhabitants compared to females, younger, higher education level, richer wealth quintiles, non-ST, and urban inhabitants, respectively. In contrast, evidence from a study conducted in China [19] and an intervention trial conducted in Nepal [20] reported an increasing trend in hypertension status awareness with the advancing age of individuals. However, previous studies from multiple LMICs also indicate that populations having low education and socioeconomic status (SES) have reduced awareness of their hypertension status, although, in Bangladesh, education was protective against a lack of awareness of the actual hypertension status [21].

Availability of health insurance influences an individual’s decision to seek treatment for their health condition, a finding consistent with our study that corroborates prior evidence suggesting those without health insurance had lower odds of availing treatment for hypertension [19]. Furthermore, a higher proportion of men compared to women were not on BP-lowering medication, a finding consistent with NFHS-4 (2015-2016) [22]. Similar to previous studies, this study's findings also suggest that older adults [23], males [20], and obese/overweight individuals [23] were less likely to attain optimal BP due to biological risk. The waist-to-hip ratio is also now emerging as a better correlate for developing both hypertension and suboptimal BP control when on medication [24]. Consequently, patients with diabetes experience greater challenges in achieving BP control due to the high prevalence of obesity and/or high waist-to-hip ratio in these comorbid patients [25].

In this study, low education was a predictor of poorly controlled hypertension. There is growing recognition that an educational gradient predisposes individuals with a lower educational level to a higher risk of onset and progression of cardiovascular disease due to improper health-seeking behavior and poor medication adherence [26].

Northeastern states of India have the highest prevalence of hypertension [27]. We also found that most states in the northeastern region of India had poor treatment-seeking behavior and poor BP control, which also correlated with their low health index. Strengthening primary health systems in low-resource settings may translate into an effective treatment cascade for hypertension care in India.

Our study has certain important public health policy implications. First, a large subset of the population in India remains undiagnosed with hypertension indicative of a lack of effective screening and missed opportunities in primary care outpatient settings despite policy directives in this regard. Additionally, screening of adolescents and young adults must be initiated as part of the medical routine as a greater proportion of these subgroups tend to remain unaware of their hypertensive status and have poor treatment-seeking behavior [22]. Patients with risk factors such as those with a higher waist-to-hip ratio should be prioritized for screening as they have an increased risk of remaining undiagnosed. Physicians should provide an enhanced focus on individuals with comorbidities such as diabetes who are less likely to have control over their BP levels, which further accelerates their risk of disease progression. Greater advocacy is needed in the National Program for Noncommunicable Diseases (NCDs) prevention in India to meet the modified strategies related to prevention and behavior change [28]. Second, six in 10 patients despite having awareness of a hypertension diagnosis are not initiated on treatment suggestive of poor treatment-seeking behavior, signifying the requirement for appropriate educational and behavioral interventions from the time of initial diagnosis. Third, there has been a significant improvement in the proportion of patients on antihypertensive treatment (40.7%) compared to the previous NFHS-4 (2015-2016; 32.6%) round suggestive of improved drug accessibility that could be secondary to schemes such as the Pradhan Mantri Jan Aushadi Yojana (PMJAY) that promote people’s access to high-quality generic medicines at affordable prices [29]. Finally, India’s health performance index does not correlate with core elements of the hypertension treatment cascade, signifying optimal maternal and child health indicators are not an appropriate proxy for the effectiveness of NCDs management that requires the incorporation of specific and relevant parameters.

There are certain limitations of this study. First, NFHS does not include the geriatric population. However, analysis from a large population data set also reflects a suboptimal treatment cascade among the elderly in India with similar loss of hypertension care at multiple stages, including measurement of hypertension (72.5%), diagnosis/awareness (57.3%), on treatment (50.5%), and control (27.5%) albeit comparatively better than younger age groups as observed in our analysis [30]. Second, the information on adherence to antihypertensive treatment, which is a key determinant of BP control was unavailable and could not be estimated in this analysis. Third, the survey did not assess the physical activity of the individuals, and therefore, we could not assess its association with BP control. Fourth, clinical diagnosis of hypertension was not established in the NFHS surveys and only reflects a statistical estimate of the surveyed population.

## Conclusions

The hypertension control cascade in younger and middle-aged groups has major lacunae at every stage, from screening and diagnosis to initiation of effective antihypertensive treatment and attainment of safe BP levels although significant improvements were observed in the screening yield and initiation of antihypertensive treatment. Identification of high-risk groups for opportunistic screening, implementation of community-based screening, strengthening primary care, and sensitizing associated practitioners are urgently warranted.
